# Antiferromagnetic Chains in a Monolayer of Molecular Qubits Assembled on Graphene

**DOI:** 10.1002/smll.73217

**Published:** 2026-04-02

**Authors:** Fabio Santanni, Matteo Briganti, Leonardo Tacconi, Marta Albanesi, Niccolò Giaconi, Andrea Luigi Sorrentino, Alessandro Veneri, Edwige Otero, Giuseppe Cucinotta, Stiven Forti, Antonio Rossi, Camilla Coletti, Giulia Serrano, Lorenzo Poggini, Roberta Sessoli, Matteo Mannini

**Affiliations:** ^1^ Department of Chemistry ‘Ugo Schiff’ DICUS, and INSTM Research Unit University of Florence Sesto Fiorentino Italy; ^2^ Synchrotron SOLEIL L'Orme des Merisiers Saint‐Aubin France; ^3^ Center for Nanotechnology Innovation @NEST Istituto Italiano di Tecnologia Pisa Italy; ^4^ Department of Industrial Engineering DIEF, and INSTM Research Unit University of Florence Florence Italy; ^5^ Istituto di Chimica Dei Composti OrganoMetallici (ICCOM) Consiglio Nazionale Delle Ricerche (CNR) Sesto Fiorentino Firenze Italy

**Keywords:** 1D antiferromagnetic coupling, 2D self‐assembled materials, copper, DFT, graphene, molecular magnetism, qubit, STM, X‐ray spectroscopy

## Abstract

The neutral Cu^2+^ complex [Cu(dttt)_2_], in which dttt^−^ is the 1,3,2‐dithiazole‐4‐thione‐5‐thiolate ligand, is a promising molecular spin qubit where a hydrogen‐free and sulfur‐rich scaffold has been designed to enhance the spin coherence. In bulk, the structural organization induces strong intermolecular antiferromagnetic exchange couplings up to about 100 cm^−1^, mediated by van der Waals interactions and propagated along 1D chains of molecules within the crystal structure. Here, the deposition by sublimation in ultra‐high vacuum conditions of [Cu(dttt)_2_] on a graphene surface is studied, focusing on investigating the topology and magnetism of ultrathin films. These deposits are characterized by combining X‐ray photoelectron spectroscopy and scanning tunneling microscopy; the latter indicates an ordered chain‐like arrangement of the assembled monolayer. Synchrotron‐based X‐ray absorption techniques flanked by density functional theory and wavefunction‐based simulations confirm the molecular ordering. These reveal that the magnetic coupling observed in bulk is also present at the monolayer level, highlighting the persistence of a 1D antiferromagnetic intermolecular coupling of about 50 cm^−1^ with a non‐negligible contribution coming from a through‐surface exchange path.

## Graphical abstract

This work explores the deposition onto graphene of a dithiazole‐based molecular qubit, [Cu(dttt)_2_], (dttt^−^ = 1,3,2‐dithiazole‐4‐thione‐5‐thiolate), under ultra‐high vacuum conditions. Analytical techniques, including STM, XPS, and X‐ray absorption, reveal the molecular arrangement and magnetic interactions, emphasizing the role of vdW interactions in the achievement of 1D AFM spin chains on graphene.

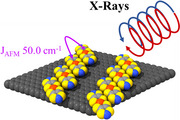

## 1 Introduction

In the quest for new materials for quantum information processing (QIP), electron‐spin‐based quantum bits, or qubits, based on magnetic molecules are promising candidates [1, 2, 3, 4, 5, 6]. Molecular qubits denote considerable and tunable structural and physical properties [5, 7]. Multi‐qubit architectures suitable for quantum logic gates implementation are achievable by chemical strategies, opening the possibility of precise tuning of qubit‐qubit magnetic exchange interactions necessary for entanglement generation [8, 9, 10, 11, 12, 13]. In particular, antiferromagnetic (AFM) materials [14] have recently gained increasing interest due to their peculiar properties in spin transport [15], including faster spin dynamics, higher propagation velocity of magnetic waves, resistance to external perturbations, and the possibility to manipulate the AFM coupling by optical means. Chains of spins 1/2 were proposed as quantum communicators [16] and are attracting an increasing interest due to recent advances in the on‐surface synthesis of 1D carbon nanostructures that carry AFM‐coupled unpaired electrons [17, 18].

To this aim, magnetic molecules offer full tuneability provided by chemical design, allowing precise control of magnetic interaction between spin centers. Realizing regular arrays of addressable entities is a crucial step toward applications in QIP and advanced experiments on single‐spin manipulation using scanning tunneling microscopy (STM) ‐based tests [19, 20, 21, 22, 23]. In this context, neutral and thermally stable molecular qubits are highly desirable for surface deposition experiments under ultra high vacuum (UHV) conditions.

**FIGURE 1:**
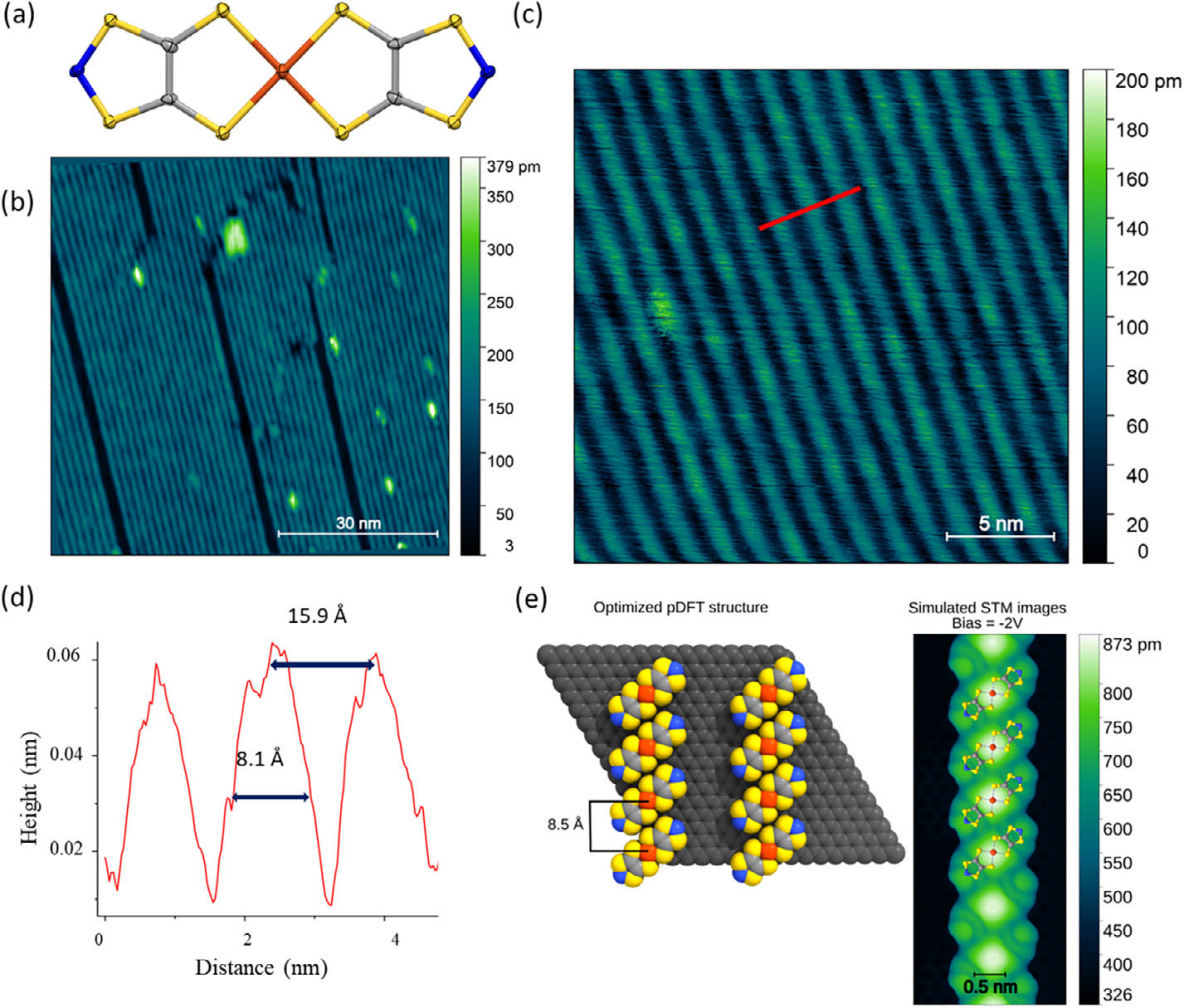
(a) Molecular structure of **Cudttt**. Color code: C, grey; N, blue; S, yellow; Cu, orange. (b,c) STM images (*T* = 35 K) of a monolayer deposit (**ML**) of **Cudttt** on graphene. 75 × 75 nm (b) and 25 × 25 nm (c); *I_t_* = 2 pA, *V_b_* = 2 V. (d) Line profile taken across the red line in panel (c). (e) Optimized **Cudttt@graphene** structure by pDFT and simulated STM image at −2.0 V (filled states).

Some of the authors have previously employed an unusual mono‐negatively charged sulfur‐rich ligand, i.e., the hydrogen‐free ligand 1,3,2‐dithiazole‐4‐thione‐5‐thiolate, dttt hereafter [24], to obtain the Cu^2+^ complex [Cu(dttt)_2_] (Figure 1a), **Cudttt** from now on [25]. An upper limit for the coherence time of ca. 300 µs has been predicted due to the nuclear spin‐depleted environment, although it is limited to 2 µs by radical impurities in the diamagnetic crystalline host. In its pure and bulk phase, **Cudttt** behaves like a 1D antiferromagnet. The exchange interaction of about 100 cm^−1^ ($J{\hat{S}}_{i}{\hat{S}}_{i+1}$ formalism) is mediated by van der Waals (vdW) interactions between S∙∙∙S donor atoms within chains of neighboring molecules.

The flat **Cudttt** molecules are also characterized by excellent stability under UHV, thus promising to realize ordered films via sublimation. Such properties make this system appealing for investigating the quantum properties of isolated molecules on the surface [21, 26]. Due to the noteworthy magnitude of intermolecular exchange coupling interactions, **Cudttt** could represent an unprecedented example of a quantum spin chain self‐assembled on the surface. To this end, we conducted an electronic, morphological, magnetic, and computational investigation of a monolayer formed by thermal sublimation on graphene. By combining STM, X‐ray photoemission spectroscopy (XPS), and synchrotron investigations with first‐principles calculations, we show that the electronic structure and magnetism of **Cudttt** are both preserved on graphene. While the study of isolated molecules is crucial for the initialisation of single qubits, the realisation of quantum gates and the transmission of information require controlled interaction between magnetic centres. In this context, the self‐assembly of **Cudttt** on graphene provides a unique platform for investigating interacting spin chains. Crucially, our findings reveal that the graphene substrate is not merely a passive support but plays an active role in promoting magnetic exchange. Our DFT calculations point out that the adsorption on graphene significantly enhances the antiferromagnetic coupling compared to the same molecular packing without the graphene surface, while preserving the integrity of the spin chains and providing a pathway to engineer robust quantum spin architectures on surfaces. The ordered layer retains the strong antiferromagnetism observed in the bulk, with a non‐negligible surface‐mediated contribution.

## 2 Results and Discussion

The molecular deposition followed the procedure described earlier for thick film growth [25], selecting here graphene on SiC as a substrate [27, 28] (see Methods for more details) and limiting the dose to a monolayer. Figure 1b,c show the STM images obtained at 35 K of the monolayer (**ML**) sample, which exhibits alternating bright and dark stripes with a periodicity of about 15.9 Å and lateral size of 8.1 Å, as reported in Figure 1d. Even if STM images do not provide sub‐molecular resolution, the size of the stripes is in line with the dimension of the **Cudttt** molecules along the axis defined by the two nitrogen atoms (11.0 Å, obtained by crystallographic data reported in ref. [25]).

To gain further insights into the well‐ordered features observed by STM, the structure of **Cudttt** chains was optimized on graphene, **Cudttt@graphene**, and the experimental STM pattern (Figure 1c) was simulated by periodic density functional theory (pDFT). A detailed explanation of the method is reported in computational details, while its validation through the modelling of high‐quality XPS spectra is reported in Section S1. Although STM clearly resolves the overall stripe‐like assemblies, precise evaluation of sub‐molecular orientation, tilt, and Cu∙∙∙Cu distances remains experimentally challenging. Indeed, our simulated STM image (Figure 1e) reveals intrachain contrast variations of only tens of picometers between densely packed molecules, justifying the observed lack of sub‐molecular resolution in our experiments. Consequently, the structural parameters and the related structure–property relationships discussed herein are inferred from the robust agreement between the experimental periodicity and our pDFT models. Based on these simulations, molecules are tilted by 40° from the propagation direction of the stripes, with the Cu∙∙∙Cu distance being 8.5 Å and the shortest S∙∙∙S distance being 3.94 Å. For comparison, the shortest S∙∙∙S distance observed in the crystal phase is 3.89 Å. This configuration maximizes the overlap between the 3*p* orbitals of sulfur with neighboring molecules, as observed in the bulk [25]. The antibonding character of the *d_x_^2^_‐y_^2^* magnetic orbital of copper delocalizes spin density on coordinated S atoms, leading to the significant intermolecular magnetic exchange interaction.

**FIGURE 2:**
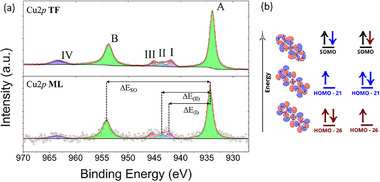
(a) Cu2*p* spectra of **Cudttt** sublimated on graphene on SiC as monolayer (**ML**, bottom) and thin film (**TF**, top). Components in magenta, blue, orange, and violet arise from shake‐up signals characteristic of the Cu^2+^ ions (I, II, III, and IV, respectively). (b) Mechanism giving rise to the simulated shake‐up peaks. The orbitals have been computed with ΔSCF method for the Cudttt^+1^ system core‐hole state, in which an electron has been removed from the Cu inner 2*p* orbitals. The red and blue colors of the isosurfaces represent the opposite phases of the wavefunctions involved in the specific ligand‐to‐metal charge transfer excitations corresponding to the satellite peaks. The orbital isosurface isovalue is 0.003e^−^/a_0_^3^.

A thorough XPS analysis was conducted on the monolayer (**ML**) and thin film (**TF** ≈ 15 nm) on graphene on SiC (see Section S1). The spectral features of both samples are well comparable with those previously observed on bulk samples and sublimated thick deposits [25], confirming that molecular integrity is preserved for **ML**. Experimental N1*s*, S2*p*, and C1*s* regions and their analyses are reported in SI (Figures S1 and S2), while we focus here on the most important Cu2*p* region (Figure 2a). The two main peaks at 933.9 eV (A) and 953.7 eV (B) are related to the Cu2*p*_3/2_ and Cu2*p*_1/2_ spin‐orbit components, respectively, with a Δ*E*_SO_ of 19.8 eV. Components at 941.9, 943.5, 945.1, and 965.5 eV arise from shake‐up signals characteristic of the Cu^2+^ ions (I, II, III, and IV, respectively) [29, 30, 31, 32, 33, 34]. Modelling of **ML** at the DFT level by delta self‐consistent field (ΔSCF) procedure (Section S1) nicely reproduces the binding energies of XPS transitions (Table S1). DFT results well reproduce the energy of satellite peaks, that arise from the promotion of inner electrons to the SOMO (Figure 2b) and are a clear indication of the Cu^2+^ oxidation state.

The experimental Cu2*p* XPS core‐level signals of **ML** and **TF** samples compared with the simulation show the absence of any significant electron transfer from the molecule to the substrate (Section S1).

Weak molecule‐substrate interactions can be better evidenced by in situ angle‐resolved photoemission spectroscopy (ARPES) experiments. Experiments performed on bare graphene and **ML** revealed that the Dirac point of pristine graphene is located at −0.44 eV (Figure S3a), while it shifts slightly to −0.46 eV in **ML** (Figure S3b). The observed variation (∼20 meV) is within the uncertainty that can arise from thermal fluctuations (comparable to the thermal energy at room temperature, ∼25 meV) and experimental noise, reinforcing the weak or negligible molecular interaction hypothesis. This is further supported by the DFT computed absorption energy, 23.18 kcal/mol, in line with what is computed for weakly interacting molecules on graphene [35, 36] and other metal surfaces [37, 38].

**FIGURE 3:**
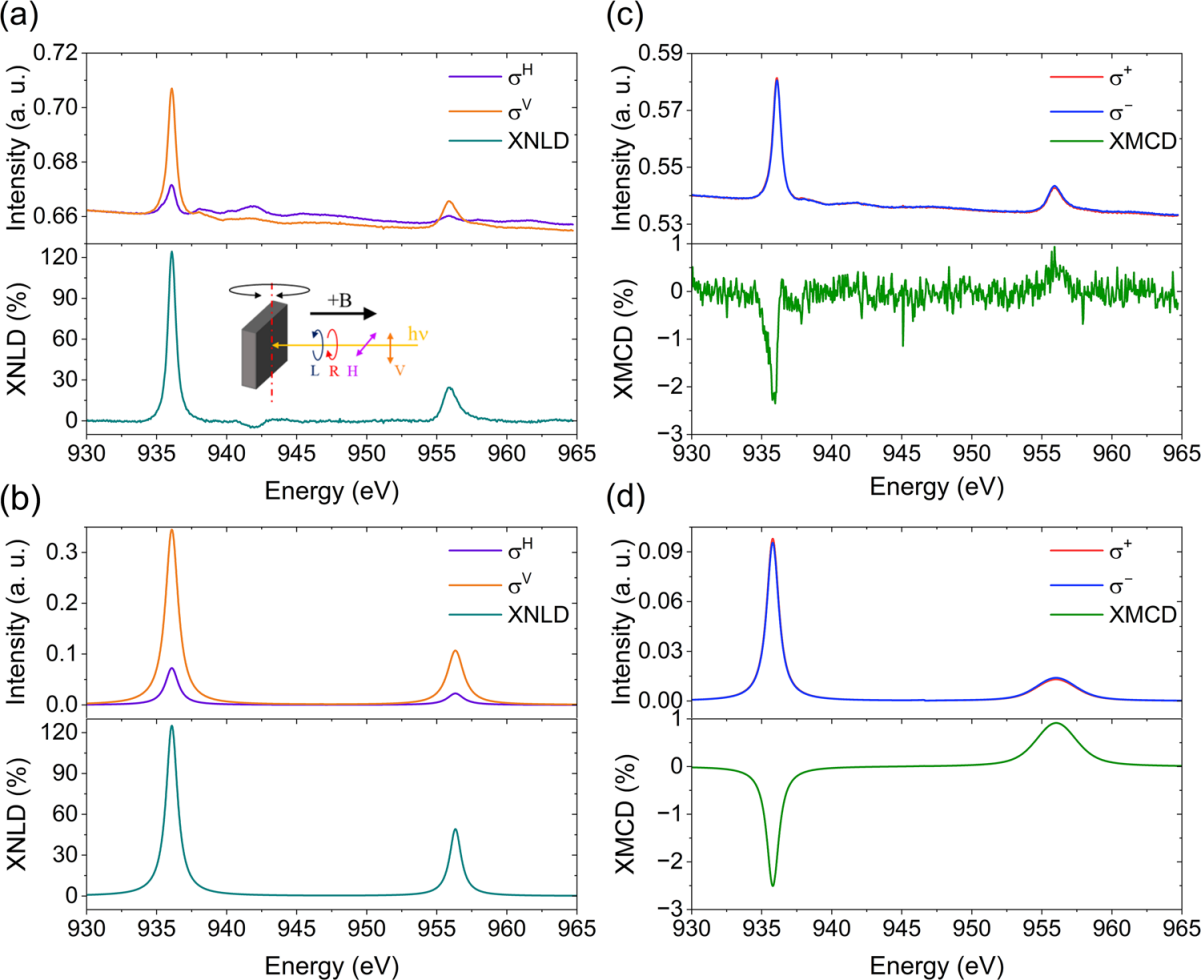
(a) XNLD measurements for **Cudttt ML** on Graphene at 1.8 K under a 60 kOe magnetic field to enhance TEY sensitivity. XNLD (dark cyan obtained from the difference of the horizontally σ^H^ and vertically σ^V^ spectra); (b) XNLD simulated spectra for **Cudttt ML** on graphene at 2.0 K; (c) XMCD spectrum measured at 1.8 K and 60 kOe. (d) simulated XAS and XMCD spectra at 1.8 K, obtained by considering the model presented in the main text.

Synchrotron experiments by X‐ray absorption spectroscopy (XAS) at the Cu L‐edge and under linearly polarized light were performed to confirm the absorption configuration suggested by STM images and pDFT simulation. The X‐ray natural linear dichroism (XNLD) signal (Figure 3a) was obtained from XAS spectra recorded at incidence angle *θ* = 45° by computing the difference between the signals collected with horizontally ($\vec{E}$ direction out of plane) and vertically ($\vec{E}$ direction in plane) polarized light. The strong XNLD compares well to what is observed with other flat‐lying molecules, e.g., Cu^2+^ phthalocyanines (CuPc) [39, 40], and it is here well reproduced in Figure 3b using the Quanty multielectron code script [41] (Section S4 and Figure S5).

The magnetic properties of **Cudttt** on the graphene substrate were investigated by combining computational methods and synchrotron techniques. Broken symmetry (BS) calculations with hybrid B3LYP functional on the final optimized structure (see computational details in Methods) estimate an intrachain AFM coupling constant of 50.0 cm^−1^. No sizeable interchain exchange interactions are computed at variance from what was observed in the more densely packed crystalline phase [25]. When we use the optimized geometry of **Cudttt@graphene** without the substrate, the intrachain exchange interaction drops to 35.9 cm^−1^. The latter value can be directly compared with that computed for the crystal phase with the same pDFT protocol, 59.1 cm^−1^. The difference can be ascribed to the slightly shorter S∙∙∙S and Cu∙∙∙Cu distances observed in the crystal. More interestingly, the significant difference between the computed exchange coupling constants with and without the substrate (14.1 cm^−1^) points to an additional AFM interaction pathway mediated by the substrate. The non‐innocent role of the substrate, originating from the overlap of the 2*p_z_* orbitals of graphene with the diffuse 3*p* orbitals of the sulfur, is proven by the partial spin density delocalized on the surface (see Figure S4).

The magnetic properties of the monolayer were then experimentally addressed by measuring XAS spectra with right (σ^−^) and left (σ^+^) circularly polarized X‐rays. The X‐ray magnetic circular dichroism (XMCD) signal is obtained as the difference (σ^+^ – σ^–^) normalized as described in Methods. The XMCD signal reported in Figure 3c was measured at normal incidence, *θ* = 0°, under an applied magnetic field of 60.0 kOe, and it reveals a main dichroic peak at 935.9 eV. The position and shape of the XMCD signal of the ML sample at the L_3_ edge resemble those of CuPc deposits in the literature [40]. However, its absolute intensity is significantly lower, approximately 3% of that observed in CuPc systems. Furthermore, the experimental XMCD percentage also deviates considerably from the value calculated for an isolated **Cudttt** molecule, being again only about 3% of the theoretical signal (see below and Figure S8). Before discussing the quantitative modelling, it is worth noting that the relatively weak intensity of the XMCD signal, even at 1.8 K and at 6 T, provides direct qualitative evidence of the strong antiferromagnetic interaction between the Cu^2+^ centres. In an ideal AFM chain, the antiparallel alignment of spins largely compensates the total magnetic moment, leaving only a residual contribution from uncompensated spins (e.g., in odd‐numbered finite chains or defects), consistent with the experimental observation.

**FIGURE 4:**
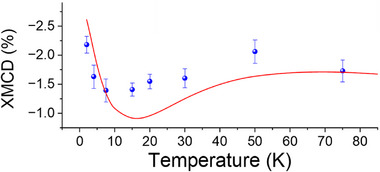
Experimental (blue dots) and simulated temperature dependence (red line) of the XMCD values at the Cu L_3_ edge at 60 kOe.

Further insights into the magnetic interactions on the surface can be gained by measuring the temperature evolution of the XMCD signal. Figure 4 and Figure S6 show the temperature behavior of the normalized dichroism at the L_3_ edge, featuring a decrease with decreasing temperature. This behavior is similar to what was observed by magnetometry in bulk **Cudttt** due to intermolecular AFM couplings [25]. Specifically, despite the low S/N ratio due to the weak magnetic signal, we notice an increase in the XMCD below 10 K, as observed in the bulk. This feature can be attributed to the finite size of chains that leads to a residual magnetic signal for odd‐membered segments, while it tends to zero for even‐membered segments and ideal infinite chains.

The correlation between the length of the molecular chains on the surface and the observed XMCD trend was evaluated by developing a simulation protocol based on a custom‐built Quanty multielectron code script [41] reproducing polarized XAS spectra at different temperatures. In this approach, XAS spectra are obtained by considering the transition probabilities between the initial and final states of the system, calculating the eigenvectors from the Hamiltonian operator describing the system. In the case of an infinite 1D magnetic chain, the exact Hamiltonian would require the introduction of an infinite number of magnetic centers. Exploiting an approach similar to numerical methods used in bulk magnetism, such as the Bonner‐Fischer model for χ(*T*) [42], XMCD spectra were simulated for closed loop‐based chains of increasing sizes, and numerical expressions were extracted depending on the number of centers in the ring. Periodic boundary conditions were applied in order to suppress boundary effects, which can be significant for small clusters (Section S4), and to obtain a smoother evolution of the XMCD signal primarily governed by the magnetic exchange interaction, as in longer antiferromagnetic segments. We simulated the XMCD spectra for rings up to 5 centers. For larger rings, the computational time becomes prohibitively high, being the basis set equal to 10*^n^*, with *n* equal to the number of centers within the ring (see Figure S7). When considering an *n*‐membered ring of antiferromagnetically coupled Cu^2+^ molecules, the initial and final states involved in the X‐ray absorption process at the Cu L_2,3_ edges can be described by the following Hamiltonian:

(1) $$\hat{H}=\sum_{i=1}^{n}{{\hat{H}}^{i}}_{ee}+{{\hat{H}}^{i}}_{SO}+{{\hat{H}}^{i}}_{CF}+{\hat{H}}_{Zeeman}^{i}+\sum_{k=1}^{n}{\hat{H}}_{ex}^{k,k+1}$$

Terms in (1) represent the interelectronic repulsion, the spin‐orbit coupling, the crystal field, the Zeeman, and the magnetic exchange interactions, respectively. The condition ${\hat{H}}_{ex}^{\left(n.n+1\right)}\equiv \;{\hat{H}}_{ex}^{\left(n.n+1\right)}$ is accounted for the ring‐like arrangement of Cu^2+^ exchanged spins (Section S4). Some restrictions were assumed to reduce the number of free parameters in the simulation procedure. The free‐ion parameters (interelectronic repulsion and spin‐orbit coupling) were taken as those calculated using the atomic Hartree‐Fock method [43, 44]. Crystal field parameters were obtained through a fitting procedure of the energy levels derived from CASSCF calculations (see Table S2 and computational details). The set of crystal field parameters is reported in Tables S4 and S5. The value of the magnetic exchange interaction constant *J_ex_* = 50.0 cm^−1^ was fixed at the value obtained from DFT (see above).

By analyzing simulated XMCD spectra as a function of temperature (Figure S8), it has been possible to extract a trend for the normalized dichroic signals at the L_3_ edge and their evolution for different sizes of *n*‐membered rings (Figure S9). [36, 45] The observed trend is consistent with the magnetic temperature behavior typically associated with even‐ and odd‐membered antiferromagnetically coupled rings; the former leading to a non‐magnetic state and the latter having one unpaired electron. The simulated temperature evolutions of the XMCD maxima for odd and even membered rings were fitted with Equations 2 and 3, respectively.

(2) $$XMCD_{odd}^{L_{3}}\;\left(T\right)=q_{1}\;\left(T\right)\cdot n_{odd}^{\beta \left(T\right)}$$

(3) $$XMCD_{even}^{L_{3}}\;\left(T\right)=q_{2}\;\left(T\right)\cdot n_{even}^{\alpha \left(T\right)}$$

For odd‐membered rings, *q*_1_(*T*) was taken as the temperature‐dependent signal of the monomer, while for even‐membered rings, *q*_2_(*T*) was taken as the temperature‐dependent signal of the dimer. Phenomenological exponents, *α*(*T*) and *β*(*T*) (Figure S10), were extracted through a fitting procedure of the simulated XMCD data as a function of ring dimension; the temperature dependence of those phenomenological exponents used in the fitting procedure is not arbitrary but reflects the underlying physics of the finite Heisenberg chains. These parameters effectively capture the competition between the thermal energy and the exchange coupling; the variation of these exponents allows the model to reproduce the deviation from a simple paramagnetic behavior across the explored temperature range.

To extract an average length n for the chains on the monolayer deposit *n_odd_* was assumed equal to *n_even_* and the temperature‐dependent experimental XMCD signal at the L_3_ edge was then fitted using the relation:

(4) $$XMCD^{L_{3}}\left(T\right)=\frac{1}{2}\left(q_{1}\left(T\right)\cdot {\langle n\rangle }^{\beta \left(T\right)}+q_{2}\left(T\right)\cdot {\langle n\rangle }^{\alpha \left(T\right)}\right)$$

The fitting procedure yielded an average length of the chain 〈*n*〉 = 14.2 ± 1.1 Cu^2+^ centers. The simulated polarized XAS and XMCD spectra at 2 K and the thermal evolution of the XMCD signal at the L_3_ edge are extracted. Despite the limited number of free variables, our model nicely reproduces all spectral features (see Figure 3d).

Moreover, as shown in Figure 4, the model reasonably reproduces the temperature trend of the dichroism. An overestimation of the signal is evident at very low temperatures, where only short odd‐membered chains possess a significant residual magnetic moment, suggesting that 〈*n*〉 is underestimated in our fitting procedure. Conversely, the model underestimates the magnetic response at intermediate temperatures and presents a more pronounced minimum compared to the experimental data. A slight deviation of *J_ex_* toward smaller values could account for these discrepancies and is likely to occur due to slight variations in the intermolecular distance, as the chain pitch is not commensurate with the graphene lattice. In this regard, we performed an *ab initio* calculation to define the strength and the origin of the observed deviation of *J_ex_*. For that purpose, a simple dimer using PBE0 functional with a def2‐VTZP basis set was used, and the *J_ex_* was evaluated using the broken symmetry approach [25]. From this test (Figure S11), it is evident that minor structural changes affect the exchange coupling interaction, reducing it from 65 to 21 wavenumbers when the Cu∙∙∙Cu distance is increased by 0.5 Å. An even worse scenario is simulated when tilting the two molecules by ten degrees, where *J_ex_* reduces by one order of magnitude. This means that the correlation along the chains can break down even if the overall structural ordering is preserved, i.e., without any apparent interruption in the STM images. Our parallel temperature‐dependent XMCD experiments and DFT simulation confirm the persistence of strong AFM interactions along chains that are defect‐free and for an average length shorter but comparable to 〈*n*〉 ≈ 50 estimated in the crystalline phase from the magnetometric data [25], thus evidencing the high degree of order achieved on the surface.

## 3 Conclusion

A comprehensive investigation of the structural, electronic, and magnetic properties of a **Cudttt** monolayer on graphene grown on SiC was carried out by coupling experimental and theoretical approaches. The XPS elemental analysis yielded results consistent with the stoichiometry and molecular scaffold with unaltered electronic structure. Similarly, the underlying graphene retains its intrinsic electronic characteristics, as evidenced by ARPES. XNLD experiments revealed that molecules lie flat on the surface. At the same time, STM topography and DFT calculations evidenced that the molecules are densely packed in well‐ordered chains with short Cu─S∙∙∙S─Cu contacts, as observed in the crystalline bulk phase. Notably, the strong AFM coupling along the chains, mediated by van der Waals contacts that characterize the bulk phase, is retained on the surface. This is confirmed by temperature‐dependent XMCD experiments. The latter, modelled in a multielectron approach, also allowed an estimation of the remarkable average length of defect‐free chain segments. DFT calculations confirmed the strength of the AFM interaction and evidenced a sizeable contribution from the graphene substrate in reinforcing the magnetic exchange through a small spin delocalization mediated by the overlap of the graphene 2*p_z_* orbitals with the diffuse sulfur 3*p* orbitals. Thus, the graphene maintains its electronic features but simultaneously enhances the AFM coupling within the **Cudttt** layer, indicating an intriguing interplay between the two materials. Future studies focusing on comparative assessments on different substrates will be crucial to fully disentangle the role of substrate's electronic structure.

**Cudttt@graphene** results in a 2D molecular material showing a through‐orbital, i.e., directional, antiferromagnetic 1D ordering which 1) takes place through vdW contacts; 2) it is the largest one reported through non‐covalent bonds; 3) it is preserved in magnitude from the bulk to the monolayer. Although of minor intensity, the magnetic exchange interaction observed in **Cudttt** is close to that observed in other antiferromagnetically coupled chains of radical spins generated in situ after deposition on Au(111) [17, 46, 47]. Covalent bonds are hard to form, break, and manipulate on the surface. Conversely, vdW contacts facilitate the molecular exchange, making it easy to tune, e.g., through an STM tip, which could allow for an unprecedented level of control at the single‐molecule level. vdW‐mediated strong magnetic interactions are relatively rare and, to the best of our knowledge, **Cudttt** represents the first molecular example of presenting such an interesting feature that can be transferred at the monolayer level. Using vdW interactions to achieve precise quantum spin chains on surfaces represents an appealing alternative to the challenging on‐surface synthesis of multiradical species. Extending this study further to manipulate the spin states in such a hybrid system could open new ways for research in the field of quantum technologies.

## 4 Methods

### 4.1 Synthesis

The synthesis of TBAdttt (TBA = tetrabutylammonium) ligand and [Cu(dttt)_2_] was performed according to the procedures reported in reference [25]. The obtained [Cu(dttt)_2_] powders were washed several times with CH_2_Cl_2_, MeOH, and Et_2_O before deposition experiments to remove unreacted precursors and contaminants (e.g., TBACl). Additional purification aimed at removing solvents and other volatile impurities was performed by thermal treatment of powders under UHV conditions. This was performed stepwise by increasing the temperature to 150 °C and waiting for the base pressure of the UHV chamber to restore.

### 4.2 Sample Preparation

Graphene was obtained via thermal decomposition of SiC(0001) [27, 28]. The process was carried out in a vertical cold‐wall reactor, where the sample was placed on a graphite susceptor and heated to ∼1300 °C for 10 min at a pressure of 750 mbar in an Ar environment. To achieve optimal coverage, the growth recipe was refined using a machine learning approach [27, 28].

A custom‐made sublimating cell equipped with a quartz crucible was employed for molecular deposition. The air‐sensitive powders were first placed in the crucible of the cell in a glove box, sealed under Ar by using a 20 cm UHV nipple equipped with an appropriate valve, then directly installed to the sublimation chamber and pumped to achieve 10^−8^/10^−9^ mbar base pressure. The crucible was heated to 385 K, keeping in the 10^−8^ mbar pressure upon the molecular sublimation. The deposition rate was directly estimated using a quartz crystal microbalance (QCM) exposed to the sublimating molecular material. Furthermore, surface coverage was validated by STM and XPS using the Cu2*p*/C1*s* ratio, which was consistent with previous findings on a similar system.

### 4.3 Scanning Tunneling Microscopy (STM)

STM was performed using an Omicron VT‐STM with an etched W tip. STM measurements were carried out at 35 K by fluxing liquid helium, with a base pressure of 10^−11^ mbar, to reduce molecular mobility on the surface.

### 4.4 X‐Ray Photoelectron Spectroscopy (XPS)

XPS data were acquired in situ using monochromatic Al Kα radiation (hν = 1486.6 eV) generated by a SPECS XR‐MS FOCUS 600 source operating at a power of 100 W (13 kV and 7.7 mA). An electron analyzer SPECS PHOIBOS 150 1DLD was mounted at 54.4° to the X‐ray source, facing the sample surface for normal emission detection. Spectra were collected under normal emission conditions with a fixed pass energy set to 40 eV. CasaXPS software was employed for spectral analysis, and calibration of all XPS spectra was performed relative to the C1*s* component of the SiC at 283.7 eV [48]. The deconvolution of the XPS spectra was carried out with a hybrid Doniach Sunjic/Gaussian–Lorentzian function and subtracting a linear background.

### 4.5 Angle Resolved Photoelectron Spectroscopy (ARPES)

ARPES data are acquired with a hemispherical analyzer (SPECS PHOIBOS 150). X‐ray photoelectron spectroscopy (XPS) spectra are obtained with a SPECS XR‐50 Al Kα X‐ray source. Angle resolved photoemission spectroscopy (ARPES) spectra are collected using He Iα radiation (21.2 eV) excitation source (SPECS‐µSirius) with a nominal spot size of 100 µm.

### 4.6 X‐Ray Absorption and Dichroic Experiments

XAS, XMCD, and XNLD investigations were performed using the DEIMOS beamline [49] at the French synchrotron facility (SOLEIL). Circular, vertical, and horizontal polarizations were utilized, and surface sensitivity was achieved through total electron yield (TEY) detection. XMCD and XNLD spectra have been calculated accordingly to the expressions:

$$\sigma_{\mathrm{XNLD}}\left(a.u.\right)=\sigma^{V}-\sigma^{H}$$

$$\sigma_{\mathrm{XMCD}}\left(a.u.\right)=\sigma^{+}-\sigma^{-}$$

and then, normalized according to:

$$\sigma_{\mathrm{XNLD}/\mathrm{XMCD}}\left(\%\right)=100\cdot \left(\sigma^{V/+}-\sigma^{H/-}\right)/\left(\mathrm{ej}\left(L_{3}\right)\right)$$

where 𝑒𝑗(L_3_) is the L_3_ edge jump of the isotropic spectrum. The **ML** sample was prepared and characterized at DICUS and transferred to the DEIMOS end‐station using a UHV suitcase (*P_base_* = 5  × 10^−10^ mbar).

### 4.7 Multiplet Ligand Field Theory Simulations

Multiplet ligand field theory simulations of polarized XAS spectra were carried out with the script language Quanty (https://www.quanty.org). The exact Hamiltonian parameters used for spectra simulations are shown in Supporting Information, Tables S4 and S5. Temperature influence was considered by weighting the transition probabilities for the Boltzmann population of each state. An energy‐dependent linewidth for the L_2,3_ edges was introduced to consider the experimental linewidths.

### 4.8 Computational Details

The CP2K 8.2 quantum chemistry software [50] was employed for all the pDFT calculations. RevPBE functional [51, 52], along with rVV10 empirical dispersion corrections, [53] were used in all geometry optimizations. Norm‐conserving Goedecker‐Teter‐Hutter pseudopotentials [54] and a double zeta basis set with polarization functions (DZVP‐MOLOPT‐SR) were employed for all the atoms. The cell parameters were kept fixed throughout the optimizations. The plane‐wave cut‐off value was set to 450 Ry. The wavefunction convergence threshold (EPS_SCF) was set to 1.0 · 10^−6^ Hartree, while the max force for the geometry optimization was set to 4.5 · 10^−3^ bohr^−1^ Hartree.

To simulate bulk properties, we employed the cell parameters and structure of the *C*2/c crystal phase. The *C*2/c cell structure of dimensions 15.695 Å × 8.439 Å × 8.306 Å and angles *α* = *γ* = 90°, *β* = 103.4° was taken as a starting point and as a reference for the bulk properties. All the cell parameters were kept fixed throughout the optimization of the bulk structure. Upon the final optimized geometry, the density of states and magnetic exchange couplings were simulated employing the B3LYP hybrid functional.

To compute exchange coupling, from the optimized crystal structure, a supercell 1 × 2 × 1 was employed. This procedure allowed us to calculate the largest exchange interaction along the b direction, the coupling that mainly determines the bulk magnetic behavior.

To simulate the monolayer properties, a 16 × 16 graphene layer was considered, for a total of 512 C atoms inside the cell. The cell dimensions were chosen to fit exactly 4 **Cudttt** molecules along the *b* axis direction, for a total of eight molecules. An orthorhombic unit cell (39.5120 Å × 34.2180 Å × 40.0000 Å, *α* = *β* = *γ* = 90°) was used throughout these optimizations. In geometry relaxations, all atomic positions were left to relax. The dimensions were chosen to ensure commensurability among the **Cudttt** intermolecular distance and the graphene lattice. These dimensions allowed including two rows of **Cudttt** molecules with 4 molecules in each.

The adsorption energy (*E_ads_*) was evaluated with the formula *E_ads_ = E_mol@surf_ – (E_mol_+E_surf_)*, where the three terms are the computed electronic energies of the eight **Cudttt** molecules adsorbed on graphene (*E_mol@surf_*), the eight molecules without the surface (*E_mol_*), and the bare graphene surface (*E_surf_*). These last two terms have been computed without further optimizations. The obtained energies have been then divided by eight to calculate the absorption energy per molecule. STM images were simulated at the pDFT level on the optimized structures according to the Tersoff–Hamann approximation as implemented in CP2K. The computed bias ranged from −3.0  to +3.0 V.

To compute the isotropic exchange couplings, the Broken symmetry approach was used, as developed by Noodleman [55], and refined for polynuclear systems [55]. The method is based on the mapping of the unrestricted Kohn‐Sham determinants on the Heisenberg Hamiltonian. This procedure requires computing the energy of the high spin state (HS), *S* = 4, and the broken symmetry state (BS) obtained by single spin flips on alternated copper atoms, for a total *S* = 0, i.e., the antiferromagnetic non‐totalsymmetric singlet. In the present case, we assume identical exchange interactions between all nearest‐neighbour molecules. Accordingly, the Hamiltonian for one chain is $\hat{H}=\sum J_{ex}{\hat{S}}_{i}\cdot {\hat{S}}_{j}$, where *i*, *j* are neighbors [Cu(dttt)_2_] molecules. Within the BS formalism, the energy difference between the high‐spin and broken‐symmetry solutions is given by *(E_HS_ − E_BS_) =* 2*J* *_ex_* *S*_1_·*S*_2_. For two *S =* 1/2 spins, this reduces to *(E_HS_ − E_BS_) = J_ex_*/2. Consequently, the energy difference between the ferromagnetic (*S =* 4) and antiferromagnetic (*S =* 0) states is  *Δ* *E* = 4*J*_*ex*_ and the coupling constants are then extracted by dividing the difference in energy by 4 [56]. Convergence to the non‐totally symmetric singlet state was enforced using the &BS section within the CP2K input, and the intended broken‐symmetry solution was confirmed by carefully checking the final spin density.

ORCA 6.0 software was employed to calculate the excited states of the systems. The multiconfigurational CASSCF calculations to obtain the ligand field parameters were performed on the isolated structure of **Cudttt**, extrapolated from pDFT optimizations on graphene. Def2‐VTZP basis set was used for all the atoms. The active space consisted of the nine electrons of Cu^2+^ in the five 3*d* orbitals, CAS (9,5). All ten states arising from the ground 2D multiplet were computed. The energy of the 3*d* orbitals was computed within the Ab Initio Ligand Field Theory (AILFT, see Table S3) [57]. ΔSCF procedure, as implemented in ORCA 6.0, was employed to compute the Cu2*p* binding energy and the excited states of the [Cu(dttt)_2_]^+^ giving rise to the satellite peaks. Def2‐VTZP basis set was also employed in this set of calculations, along with the PBE0 density functional [58].

## Conflicts of Interest

The authors declare no conflict of interest.

## Supporting information




**Supporting File**: smll73217‐sup‐0001‐SuppMat.pdf.

## Data Availability

The data that support the findings of this study are available from the corresponding author upon request.
