# Supplementary material for: Antiferromagnetic Chains in a Monolayer of Molecular Qubits Assembled on Graphene
Source: Small. 2026 Apr 2;22(27):e73217. doi: 10.1002/smll.73217 (PMC13173315; doi:10.1002/smll.73217)
Supplement: Supplementary file 1 — Supporting File: smll73217‐sup‐0001‐SuppMat.pdf. [file SMLL-22-e73217-s001.pdf]

Supporting Information

Antiferromagnetic chains in a monolayer of Molecular Qubits assembled on Graphene.

Fabio Santanni, Matteo Briganti, Leonardo Tacconi, Marta Albanesi, Niccolò Giaconi, Andrea Luigi Sorrentino, Alessandro Veneri, Edwige Otero, Giuseppe Cucinotta, Stiven Forti, Antonio Rossi, Camilla Coletti, Giulia Serrano, Lorenzo Poggini,* Roberta Sessoli, Matteo Mannini*

Table of contents

S.1. Detailed XPS discussion 2
S.2 ARPES characterization 5
S.3 Computational results 6
Periodic DFT results 6
CASSCF results 6
S.4 XAS results and simulations 6
Simulation of polarized XAS spectra 7
Fitting of crystal field parameters 8
Simulated X-ray natural linear dichroism spectra 9
Experimental XMCD vs temperature 10
Computational time needed to simulate XMCD spectra of n-membered rings 10
Simulated X-ray magnetic circular dichroism spectra 11
Thermal evolution of the simulated XMCD signal at L3 edge 13
Phenomenological model for the thermal evolution of the XMCD signal at L3 edge for even and odd-membered rings 13
References 14

S.1. Detailed XPS discussion

XPS analysis has been carried out to gain further insight into the molecular/graphene interaction by comparing the ML on graphene on SiC with a thin film (TF) of about 15 nm (Figure 2). The spectral features of both samples closely resemble those previously observed in bulk phase samples and sublimated thin deposits.[1]

The core-level XPS N1*s* region has a main component at 399.8 eV and a shake-up feature at 401.4 eV (Figure S1a);[1] the XPS S2*s* region, Figure S1b, has two equally intense components at 226.9 and 229.4 eV; a third small component weighting less than 3% of the whole sulfur contribution appears in the deposit and can be attributed to oxidized sulfur.[1]

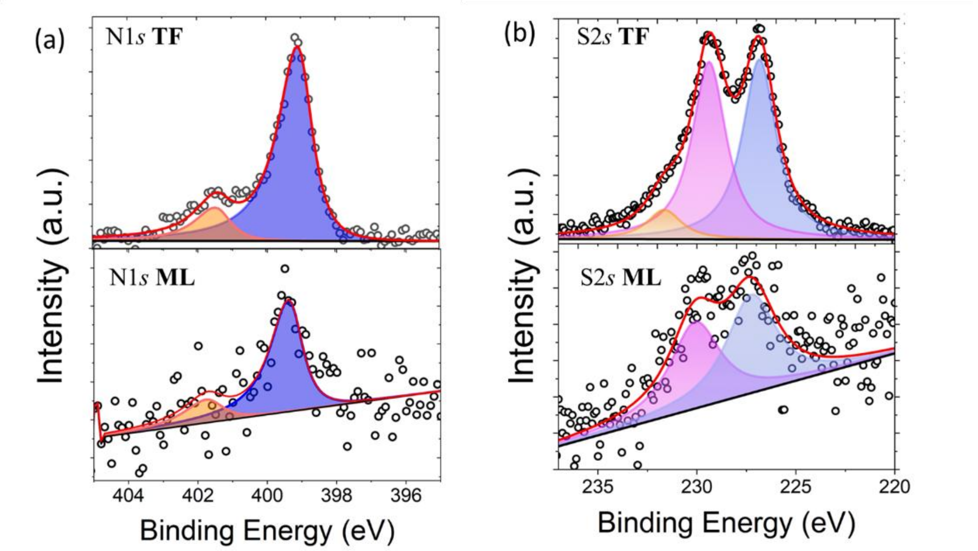

Figure S1. a) $N1_s$ and b) $S2_s$ XPS spectra of **Cudttt** sublimated on graphene on SiC as monolayer (**ML**, bottom) and thin film (**TF**, top). A linear background was subtracted from both spectra for clarity.

A contribution in the C1*s* region attributable to the **Cudttt** is hidden by the intense graphene signal in the **ML** sample, while it emerges at 285.9 eV expected for the C−S bonds[S1,S2] in the **TF** sample (see Figure S2). The S2*p* peak of the **ML** sample is hidden under the Si2*p* peak from the Si-C substrate supporting the graphene layer, making it unsuitable for both the investigation of the sulphur chemical state and semiquantitative analysis. Figure S2 also reports the bare graphene, revealing the characteristic C1*s* spectrum of graphene on SiC substrate, with the most intense peak at 283.7eV representing the SiC contribution.[S3] The graphene-related peak is at 284.6 eV; two surface components (S1 and S2) are related to the carbon-based buffer layer underneath the graphene.[S4] The

S1/S2 peak area ratio is in line with the number of C atoms covalently and not covalently bonding to the SiC substrate.

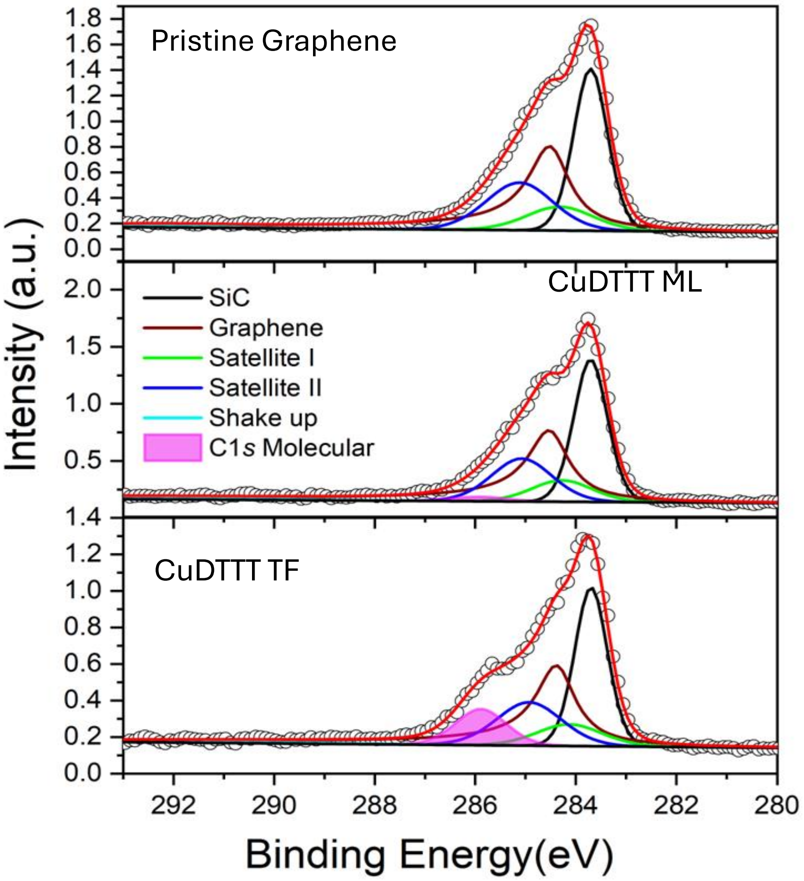

Figure S2. C1*s* region of pristine Graphene (top), **ML** (middle) and **TF** (bottom). A linear background is used.

The main peaks in the Cu2*p* region (Figure 2a of main text) lie at 933.9 eV (A) and 953.7 eV (B) and are related to the Cu2$p_{3/2}$ and Cu2$p_{1/2}$ spin-orbit components, respectively, with a DESO of 19.8 eV.[6] Components at 941.9 eV, 943.5 eV, 945.1 eV, and 965.5 eV arise from shake-up signals characteristic of the Cu^{2+} ions (I, II, III, and IV, respectively).[6–11] A more accurate comparison between the **ML** and **TF** samples reveals a shift of about 0.35 eV to higher binding energy in the monolayer with respect to the thin film. This suggests a possible partial electron transfer from the molecule to the surface or a variation in the local interface dipole and electron screening, thus originating a shift in the XPS components.[12,13] The dielectric screening effect of the underlying graphene substrate can also contribute.[13] Among these possibilities, screening effects are supported by the absence of significant variation of the energy distance ΔE(I) and ΔE(II) between the **TF** and **ML**: their values go from 7.9 eV in the **TF** to 8.0 eV in the **ML** sample and from 9.6 eV in the **TF** sample to 9.5 eV in the **ML** sample, respectively.

The XPS spectra from the **TF** to the **ML** provide a high-quality data set to benchmark our DFT protocol. Therefore, the XPS binding energies were modelled at the DFT level by delta self-consistent field (ΔSCF) procedure (see computational details), which allows the computation of the energies and wavefunctions of states with an arbitrary occupation of the Molecular Orbitals to take into account also the Cu2*p* shake-up satellite peaks (see Figure 2b). ΔSCF calculations were performed on two isolated structures of **Cudttt**, the crystallographic geometry ($[Cu(dttt)_2]_{Cryst}$), and the one extrapolated from pDFT optimization on graphene (**Cudtttopt~~@graphene~~**) to simulate the binding energy of the main Cu2*p* peak and the satellites (Table S1). Experimental and calculated delta values are in good agreement. Notwithstanding the DFT method overestimates the absolute energies (but they agree with XPS spectra of gas-phase molecules with similar structures[14]), these results indicate that no significant molecular structural variations are occurring after their surface confinement. ΔSCF also nicely reproduces the energies of the satellite peaks that arise from the promotion of inner electrons to the SOMO and are a clear indication of the Cu^{2+} oxidation state. Finally, the Cu2*p* main peak was simulated in the presence of the surface, **Cudtttopt@graphene**, and, in this case, the computed Cu2*p* binding energy is 940.1 eV, and it is only moderately affected by the interaction with the substrate, accordingly to the experimental observations. All these results demonstrate that our DFT modelling consistently reproduces the electronic structure of **Cudttt** across different environments, from the crystalline phase to the adsorbed and experimental ones, thus providing a solid foundation for the accurate computation of its magnetic properties (see below). At the same time, all the ΔSCF calculations also confirm the absence of electron transfer from the molecule to the substrate, as experimentally observed (see below).

Table S1. Computed binding energies of Cu2*p* orbitals and relative shake-up peaks for $[Cu(dttt)2]_{Cryst}$, **Cudtttopt~~@graphene~~** and **Cudtttopt@graphene** compared with the experimental one obtained by XPS measures. All energies are in eV.

	**ΔSCF calculation, eV**			**Experimental XPS data, eV**	
	**$[Cu(dttt)_2]_{Cryst}$**	**$Cudttt_{opt}$@graphene**	**$Cudttt_{opt}$@graphene**	**TF**	**ML**
Cu $2p_{1/2}$	942.2	942.3	940.1	933.9	934.3
Shake-up 1	950.7	950.7	--	941.9	942.3
Shake-up 2	952.0	952.0	--	943.5	943.8
$\Delta E_{(I)}$	8.47	8.44	--	7.9	8.0

$\Delta E2_{(II)}$	9.72	9.78	--	9.6	9.5

S.2. ARPES characterization

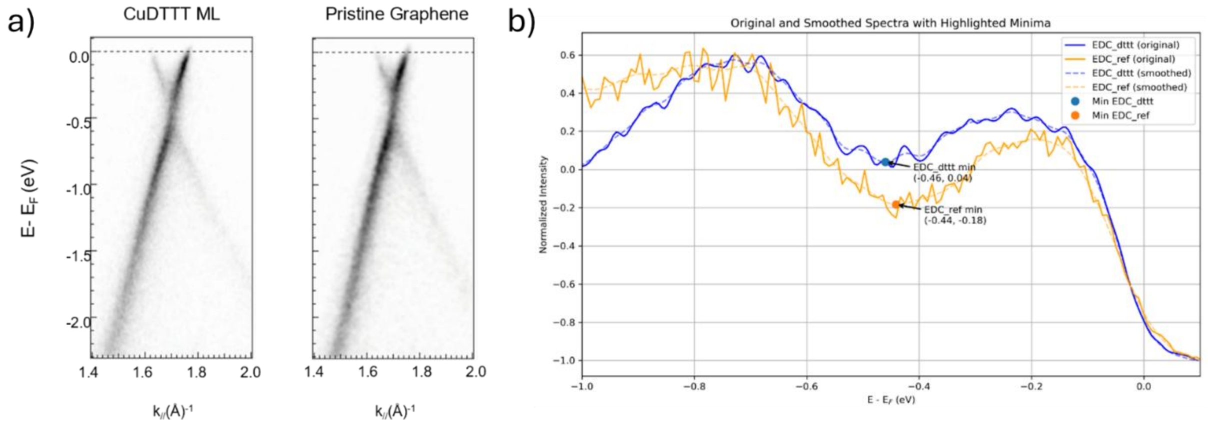

Figure S3.a) ARPES intensity of the graphene π band around k// on **ML** sample and on pristine Graphene; b) Dirac point on pristine (orange line) and on **ML** sample.

S.3 Computational results

Periodic DFT results

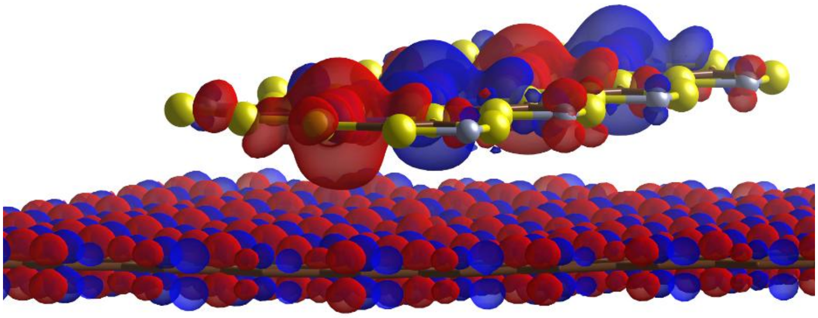

Figure S4. Depiction of the spin density difference. This is obtained by subtracting the spin density of the optimized **Cudttt** molecules on graphene and the spin density of **Cudttt** molecules without the graphene substrate below. The isosurface isovalue is 2.4e−05 e^{-}/a_0^3.

CASSCF results

Computed CASSCF states

Table S2. Energies of the five Kramers' doublets computed at the CASSCF level of theory.

**Doublet**	E_0	E_1	E_2	E_3	E_4
**Energy** (cm^{-1})	0	13903	14798	16172	16714

Table S3. Energies of the five 3*d* orbitals computed within the Ab Initio Ligand Field Theory (AILFT).

**3*d* Orbital**	z^2	xy	xz	yz	x^2-y^2
**Energy** (cm^{-1})	0	45	660	1720	15928

S.4 XAS results and simulations

Choice of the antiferromagnetic chain model

The magnetic behavior of 1D antiferromagnetic chains can be modeled by either using open boundary conditions (OBC) or periodic boundary conditions (PBC).[S15–17] For sufficiently low temperatures, i.e., when k_BT is well below the first excitation gap of the finite cluster, the magnetic response is solely governed by the ground state. Importantly, the ground state total spin is independent of the choice of boundary conditions (i.e., S = 1/2 and S = 0 for odd-and even-membered chains, respectively).[S18] In the limit in which only the ground state is thermally populated, OBC and PBC yield identical magnetic responses. By contrast, the overall energy ladder depends sensitively on the boundary conditions. Once excited states become thermally accessible, these spectral differences can lead to distinct magnetic responses at elevated temperatures. In small clusters, such as those considered in this work, edge contributions under OBC represent a sizeable correction to the magnetic response. As our objective was to approximate the behavior of longer antiferromagnetic segments using calculations restricted to small clusters, PBC were adopted as a convenient proxy to suppress edge effects and obtain a temperature dependence governed primarily by the intrinsic magnetic exchange interactions between the metal centers.

Simulation of polarized XAS spectra

Polarized X-ray absorption spectra are simulated using the script language Quanty, calculating the transition probabilities between the ground and excited states involved in the $L_{2,3}$ edge of Cu, i.e., the $2p^63d^9$ and $2p^53d^{10}$ electronic states. In both cases, the states are computed from the following general form of the Hamiltonian.

$$\hat{H} = \sum_{i=1}^{n} \hat{H}^i{}_{ee} + \hat{H}^i{}_{SO} + \hat{H}^i{}_{CF} + \hat{H}^i_{Zeeman} + \sum_{i=1}^{n}\sum_{j\neq i}^{n} \hat{H}^{ij}_{ex}$$

Specifically, the ground state Hamiltonian is described by the following terms, and the used parameters are reported in Table S2.

a. Coulomb interaction among electrons within the 3*d* shell, which is parametrized with Slater-Condon integrals F^k, where $0 \leq k \leq 2l$. For 3d electrons l is equal to 2 and *k* is then comprised between 0 and 4. Furthermore, F^0 denotes an energy shift and hence was not considered.

$$\hat{H}_{ee} = \sum_{k=2,4} F^k(r) f_k(\phi)$$

b. Spin-orbit interaction in the 3*d* shell, expressed as follows.

$$\hat{H}_{SO} = \sum_{i=1}^{9} \zeta_{3d}(r_i) \hat{l}_i \cdot \hat{s}_i$$

c. Crystal field interactions acting on the 3*d* shell. In the Wybourne's notation this can be parametrized as follows.

$$\hat{H}_{CF} = \sum_{i=1}^{9} \sum_{k=2,4} \sum_{q=0}^{k} \left[B_k^q \left(C_{-k}^q(i) + (-1)^k C_k^q(i) \right) + i B_k'^q \left(C_{-k}^q(i) - (-1)^k C_k^{q(i)} \right) \right]$$

The number of parameters, in principle equal to 13, can be reduced due to symmetry of the coordination environment. In the [Cu(dttt)$_2$] molecule, the metal center has a square planar symmetry, and the crystal field can then be modelled with four parameters: $B_2^0, B_2^2, B_4^0, B_4^4$.

d. Zeeman interaction is expressed as follows.

$$\hat{H}_Z = \sum_{i=1}^{9} \mu_B \left(\kappa \hat{l}_i + g_e \hat{s}_i \right) \cdot \vec{H}$$

e. Magnetic exchange interaction. Considering that *n*-membered rings of increasing sizes have been simulated, we used the following expression of the magnetic exchange interaction Hamiltonian.

$$\hat{H}_{ex} = J_{ex} \sum_{i=1}^{n} \hat{S}_i \cdot \hat{S}_{i+1}$$

In particular, when $I = n$ we get that $i+1 = 1$. Basically, the magnetic exchange interaction constant is $J_{ex} \neq 0$ only for neighboring spins.

Interelectronic repulsion and spin-orbit coupling parameters are obtained by Cowan's Hartree Fock (HF) program. Crystal field parameters are obtained through the fitting procedure described below. The magnetic exchange interaction constant is taken as the one calculated from ab initio calculations.

Table S4. Hamiltonian parameters used to simulate the ground state of coupled **Cudttt.** Interelectronic repulsion parameters were scaled by a factor equal to 0.8. All values are reported in eV.

F^2	F^4	ζ_{3d}	B_2^0	B_2^2	B_4^0	B_4^4	J_{ex}
12.855	7.981	0.102	1.896	-0.215	3.402	0.247	0.0062

The Hamiltonian presented above must be remodeled to consider unpaired $2p$ electrons for calculating the excited states. The interelectronic repulsion and the spin-orbit coupling are modified as follows.

$$\hat{H}_{ee} = \sum_{k=2,4} F_{3d}^k(r) f_k(\phi) + F_{2p}^2 f_2(\phi) + \sum_{k=1,3} G^k(r) g_k(\phi)$$

$$\hat{H}_{SO} = \sum_{i=1}^{10} \zeta_{3d}(r_i)\hat{l}_i \cdot \hat{s}_i + \sum_{i=1}^{5} \zeta_{2p}(r_i)\hat{l}_i \cdot \hat{s}_i$$

Excited state interelectronic repulsion and spin-orbit coupling parameters are shown in Tables S4 and S5.

Table S5. Excited state single ion parameters for Cu^{2+} taken from the literature. Interelectronic repulsion parameters were scaled by a factor 0.8. All values are expressed in eV.

F_{3d}^2	F_{3d}^4	F_{2p}^2	G^1	G^3	ζ_{3d}	ζ_{2p}
13.612	8.458	8.178	6.170	3.511	0.124	13.499

The spectra are simulated as the sum of all possible transitions, with each transition weighted by the corresponding Boltzmann population to account for thermal energy. Due to the huge number of levels in an n-membered ring system, the summation is restricted only to a few levels. We considered 10, 100, 1000, 100, and 64 states for the monomer, dimer, trimer, tetramer, and pentamer, respectively.

The crystal field surrounding the copper ion was modeled as a square-planar field, described by B_2^0, B_4^0 and B_4^4 parameters, with an additional in-plane distortion breaking fourfold symmetry, represented by a B_2^2 term. It is noteworthy that other terms compatible with C_2 symmetry, like B_4^2, could be included in the Hamiltonian. Nonetheless, the inclusion of an additional term does not lead to any

significant difference either in the electronic structure or the simulated XMCD signal for a single copper ion.

Fitting of crystal field parameters

A fitting procedure of the crystal field parameters was conducted using a custom-made program combining Quanty and a MATLAB script. The energy levels are calculated exploiting Quanty, while the error is calculated by considering energy levels from *ab initio* results using the following equation.

$$err = \sqrt{\frac{\sum_{i=1}^{m}(X_i - Y_i)^2}{m}}$$

Where X_i represents the energies obtained with Quanty and Y_i from *ab initio* calculations. A MATLAB script based on the fMINUIT (https://elly2023.smfi.unipr.it/course/view.php?id=250#section-3), a suite of minimization tools ported from MINUIT[S5], allowed us to extract the best crystal field parameters and reproduce the ab initio energies.

Simulated X-ray natural linear dichroism spectra

XNLD spectra were simulated for a single **Cudttt** molecule adsorbed over graphene in a planar configuration and for a dimer. As the magnetic features do not contribute to the XNLD signal, the simulated spectra were identical for the monomer and the dimer. As observed, the intensity of the normalized simulated spectrum closely reproduces the experiment, further confirming how molecules are adsorbed over graphene in a flat-ordered lattice.

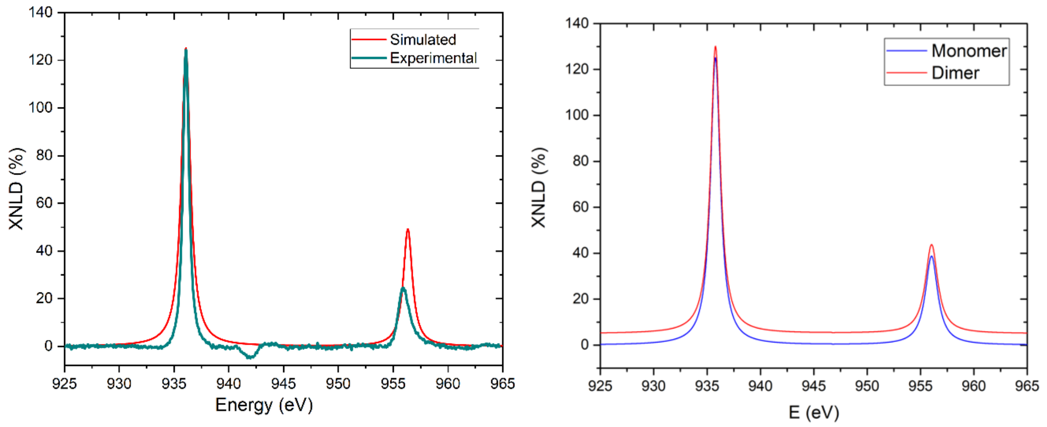

Figure S5. Comparison between the experimental and simulated XNLD spectra for the monomer (left). Comparison between the simulated spectra of the monomer and of the dimer (right).

Experimental XMCD vs temperature

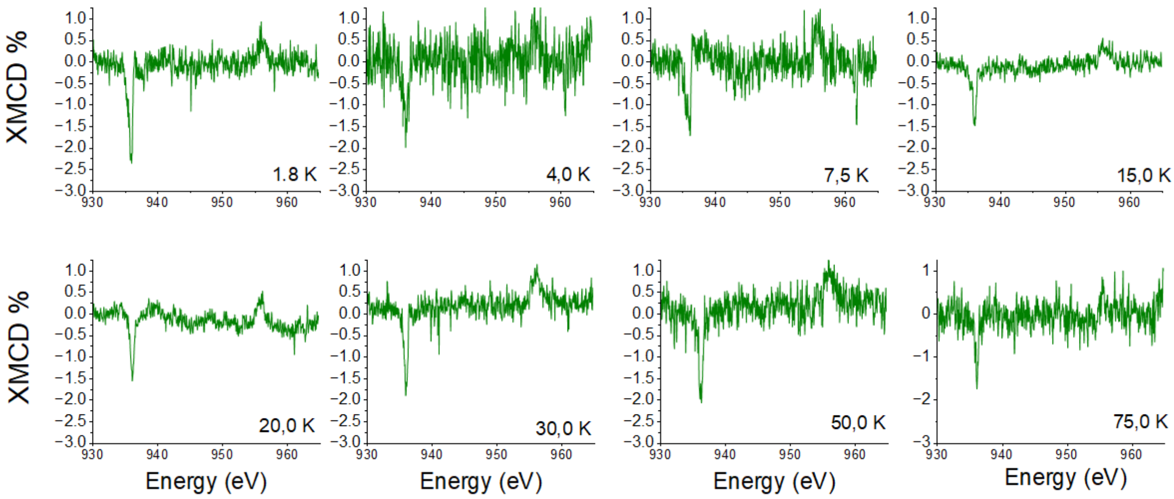

Figure S6. Experimental XMCD spectra acquired between 1.8 K and 75.0 K.

Computational time needed to simulate XMCD spectra of n-membered rings

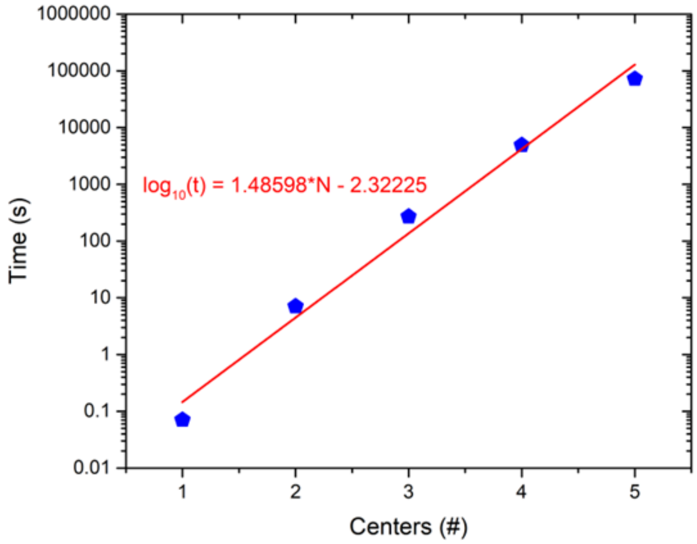

Figure S7. Simulation time needed to simulate one XMCD spectra for an *n*-membered ring (blue dots). The red line is the fitted linear trend of time as a function of the centers' number. Following this trend, the time required to simulate one spectrum for a hexamer is about forty-five days.

Simulated X-ray magnetic circular dichroism spectra

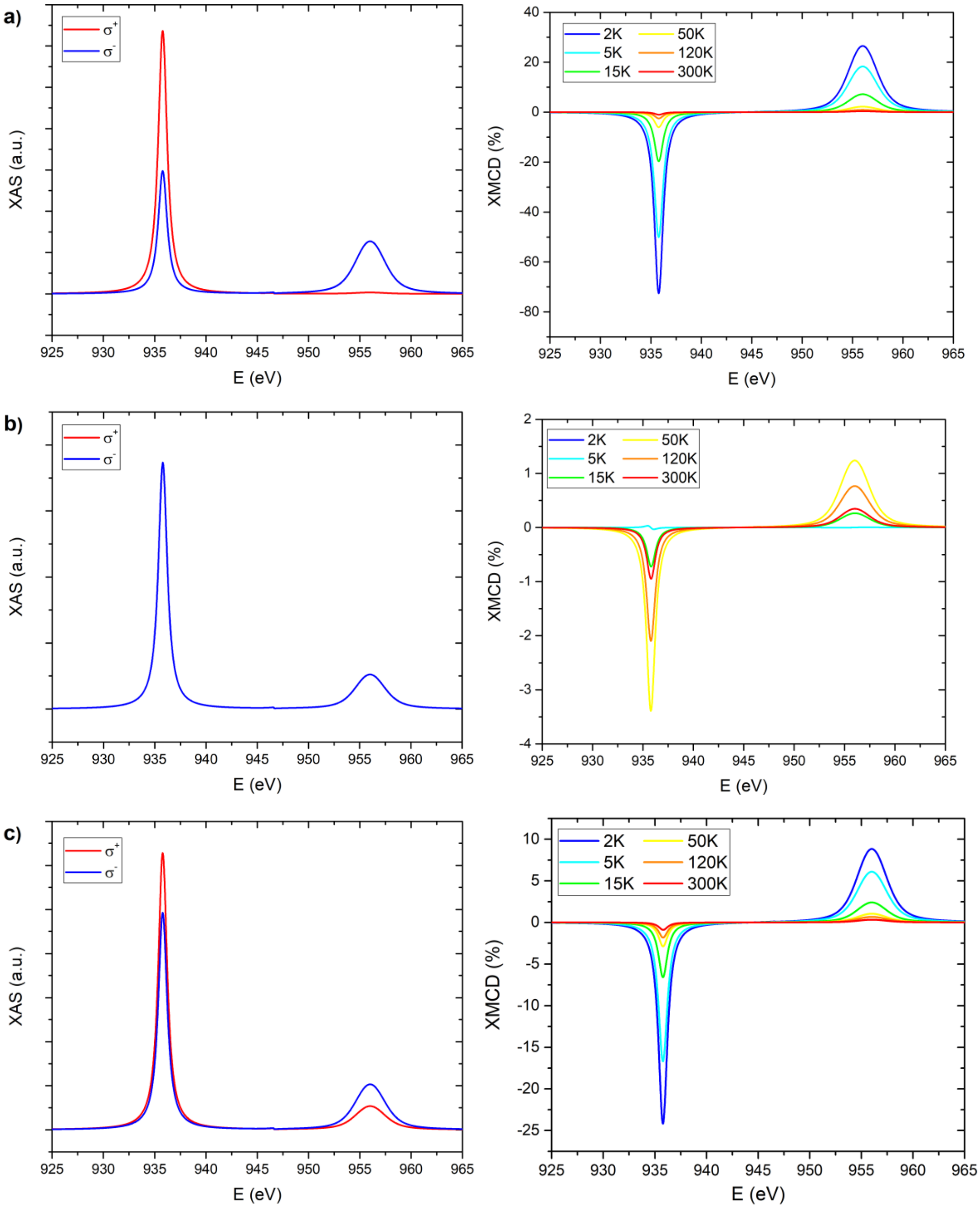

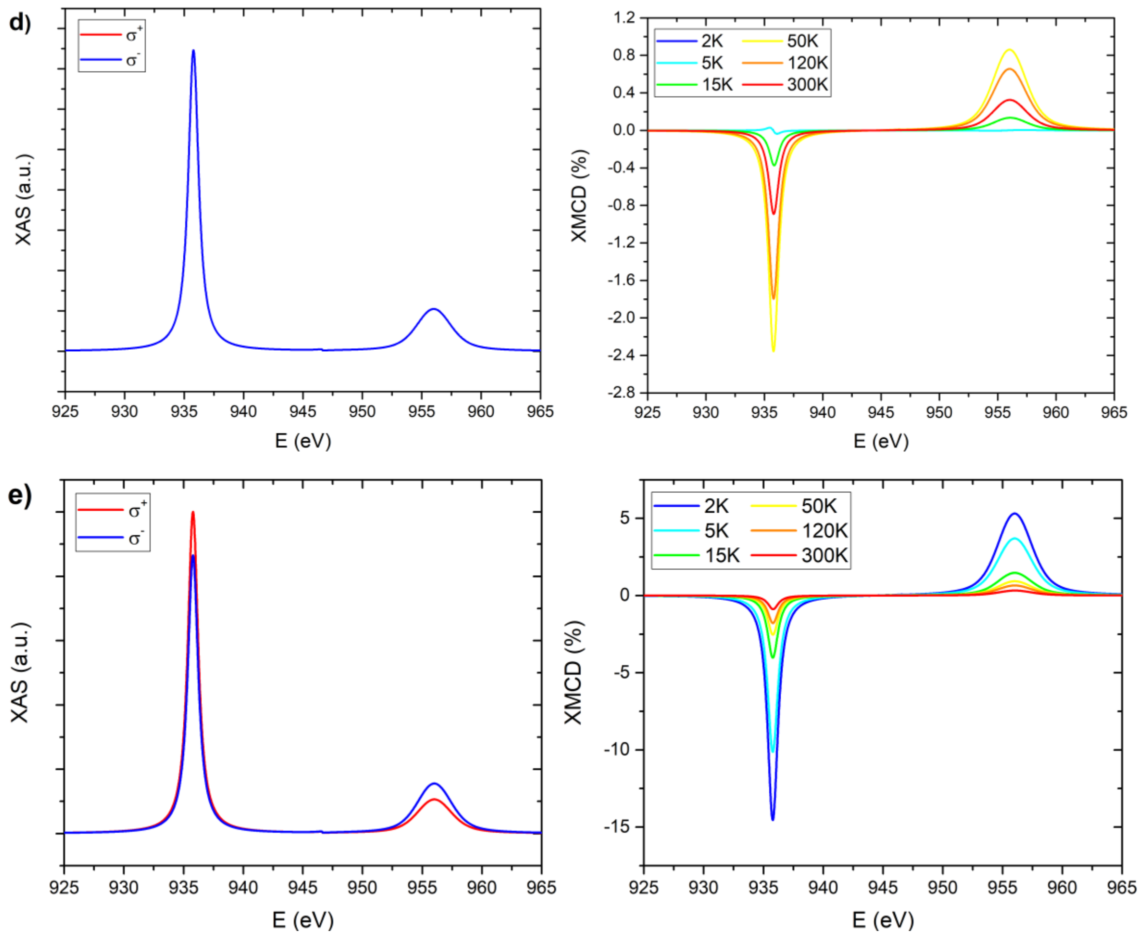

Figure S8. Simulated XAS spectra with circularly polarized light at 2 K (left) and XMCD spectra at various temperatures (right). Simulations on a) monomer; b) dimer; c) trimer; d) tetramer; e) pentamer.

Thermal evolution of the simulated XMCD signal at L3 edge

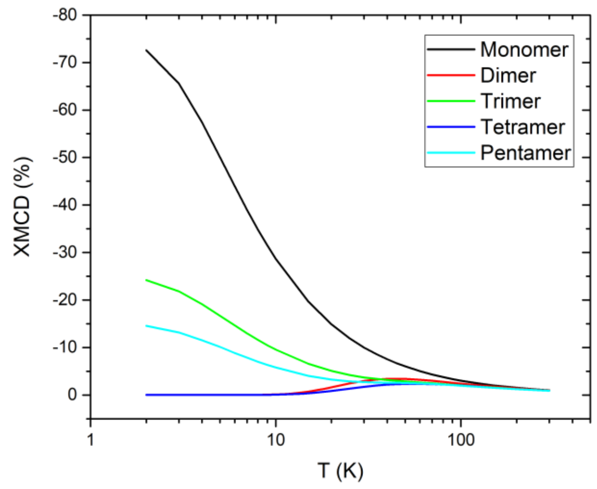

Figure S9. Normalized XMCD signal extracted from simulations at the Cu^{2+} L_3 edge.

Phenomenological model for the thermal evolution of the XMCD signal at L_3 edge for even and odd membered rings

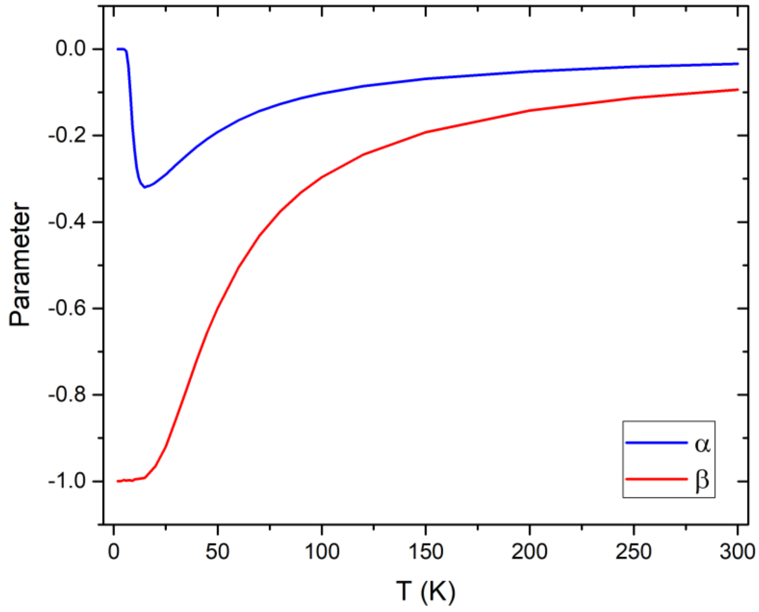

Figure S10. Thermal evolution of the phenomenological exponents α and β as discussed in the main text.

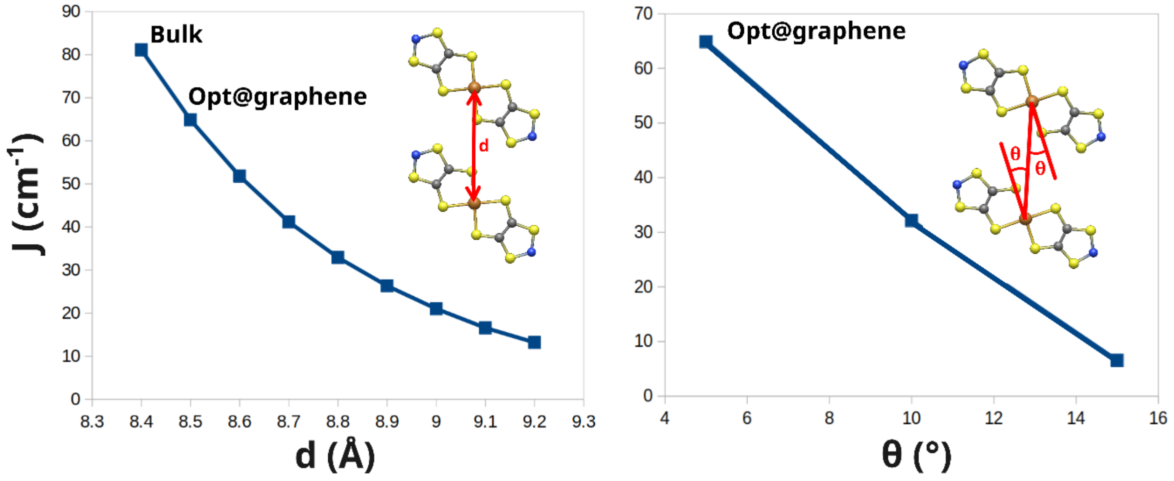

Figure S11. Computed exchange coupling constant as a function of Cu···Cu distance (left panel), and the rotation of the molecules with respect to the Cu-Cu axis (right panel). We used the PBE0 functional with a def2-VTZP basis set, while the exchange coupling was evaluated using the broken symmetry approach, as described in ref. [S1].

References

[S1] F. Santanni, M. Briganti, G. Serrano, E. Salvadori, A. Veneri, C. Batistoni, S. F. Russi, S. Menichetti, M. Mannini, M. Chiesa, L. Sorace, R. Sessoli, *JACS Au* **2023**, *3*, 1250.

[S2] L. Hohmann, K. Marks, T.-E. Chien, H. Öström, T. Hansson, M. Muntwiler, K. Engvall, M. Göthelid, D. J. Harding, *J. Phys. Chem. C* **2024**, *128*, 67.

[S3] J. Ossowski, G. Nascimbeni, T. Żaba, E. Verwüster, J. Rysz, A. Terfort, M. Zharnikov, E. Zojer, P. Cyganik, *J. Phys. Chem. C* **2017**, *121*, 28031.

[S4] K. V. Emtsev, A. Bostwick, K. Horn, J. Jobst, G. L. Kellogg, L. Ley, J. L. McChesney, T. Ohta, S. A. Reshanov, J. Röhrl, E. Rotenberg, A. K. Schmid, D. Waldmann, H. B. Weber, T. Seyller, *Nat. Mater.* **2009**, *8*, 203.

[S5] S. Forti, U. Starke, *J. Phys. D. Appl. Phys.* **2014**, *47*, 094013.

[S6] K. Okada, J. Kawai, A. Kotani, *Phys. Rev. B* **1993**, *48*, 10733.

[S7] A. D. Fedorenko, L. N. Mazalov, I. M. Oglezneva, E. Y. Fursova, V. I. Ovcharenko, *J. Struct. Chem.* **2016**, *57*, 1121.

[S8] L. N. Mazalov, G. I. Semushkina, S. A. Lavrukhina, E. V. Korotaev, A. I. Boronin, R. V. Gulyaev, T. V. Basova, *J. Struct. Chem.* **2012**, *53*, 1046.

[S9] M. Mosaferi, P. Selles, T. Miteva, A. Ferté, S. Carniato, *J. Phys. Chem. A* **2022**, *126*, 4902.

[S10] H. Ikeno, F. M. F. de Groot, E. Stavitski, I. Tanaka, *J. Phys. Condens. Matter* **2009**, *21*, 104208.

[S11] J. Conradie, E. Erasmus, *J. Electron Spectros. Relat. Phenomena* **2022**, *259*, 147241.

[S12] T. C. Taucher, I. Hehn, O. T. Hofmann, M. Zharnikov, E. Zojer, *J. Phys. Chem. C* **2016**, *120*, 3428.

[S13] L. Poggini, A. L. Sorrentino, D. Ranieri, A. Calloni, F. Santanni, N. Giaconi, G. Cucinotta, E. Otero, D. Longo, B. Cortigiani, A. Caneschi, G. Bussetti, R. Sessoli, M. Mannini, G. Serrano, *Adv. Phys. Res.* **2024**, *3*, 2300121.

[S14] J. S. H. Q. Perera, D. C. Frost, C. A. McDowell, *J. Chem. Phys.* **1980**, *72*, 5151.

[S15] J. C. Bonner, M. E. Fisher, *Phys. Rev.* **1964**, *135*, A640.

[S16] U. Schollwöck, *Rev. Mod. Phys.* **2005**, *77*, 259.

[S17] J. Borysowicz, T. A. Kaplan, P. Horsch, *Phys. Rev. B* **1985**, *31*, 1590.

[S18] E. Lieb, T. Schultz, D. Mattis, *Ann. Phys. (N. Y).* **1961**, *16*, 407.

[S19] F. James, M. Roos, *MINUIT: Function Minimization and Error Analysis Reference Manual*, **1975**.
